# Presentation and management of urethral erosion caused by mid-urethral sling

**DOI:** 10.1007/s11845-025-03949-w

**Published:** 2025-04-28

**Authors:** Olwyn E. Lynch, Eabhann M. O’Connor, Bianca Barea, James C. Forde

**Affiliations:** Department of Urology, Beaumont Hospital, Dublin 9, Ireland

**Keywords:** Erosion, Mesh, Mid-urethral sling, Polypropylene, Sling

## Abstract

**Background:**

Mid-urethral slings (MUS) have been the standard of care in surgical management of female stress urinary incontinence (SUI) internationally. Complications including pain and erosion has led to a temporary “pause” of their use in the UK and Ireland. We report on our experience to date on patient presentation, operative management, and post-operative outcomes in management of MUS erosion.

**Methods:**

Review of female patients who had partial MUS removal due to urethral erosion over a 7-year period. Data on patient presentation, operative technique, and post-operative outcomes were collected and collated.

**Results:**

A total of 21 patients were identified. Overall, 66% of patients presented with symptoms of urinary tract infection (UTI). Other presentations include overactive bladder symptoms (52%), recurrent incontinence (42%), or dyspareunia (9%). Some patients presented with a combination of these symptoms. Overall, 9 patients had a trans-obturator tape (TOT), 6 had a trans-vaginal tape (TVT), and 4 had unspecified type of MUS. Two patients had 2 previous MUS insertions.

Initial operative management with urethroscopy and laser fragmentation in cases with significant MUS calcification was required in 18% (*n* = 4) of patients. A total of 86% (*n* = 18) of patients had urethral erosion that required formal urethral repair and a short period with an indwelling catheter.

All patients had resolution of their UTI symptoms post procedure. Fifteen patients required further surgical intervention to manage recurrent incontinence after MUS removal.

**Conclusion:**

Partial removal of MUS due to urethral erosion improves patient symptoms. However, the majority have recurrence of SUI and require further intervention.

## Introduction

The management of stress urinary incontinence (SUI) in the UK and Ireland has changed significantly over the last number of years. Until 2018, the standard of care in the operative management of SUI was with the insertion of minimally invasive polypropylene mid-urethral sling (MUS) [1] via either a trans-vaginal (TVT) or trans-obturator (TOT) route [2].

Unfortunately, a small proportion of women have experienced significant complications following MUS insertion [1]. Urethral MUS erosion may result in significant morbidity and can present with a myriad of symptoms including worsening lower urinary tract symptoms, dyspareunia, and UTIs [3]. Despite the low incidence of urethral erosion, the resultant complications can be significant and typically necessitate operative intervention. Moreover, patients will often require subsequent corrective operations for their resultant worsening or new SUI.

MUS removal following urethral erosion is a relatively uncommon operative undertaking. Evidence in the literature remains sparse with small population case series using a variety of techniques [3, 4]. Consensus is lacking on optimal surgical management of these challenging cases. In this study, we report on our experience with regard to patient presentation, operative management, post-operative outcomes, and follow-up.

## Materials and methods

Institutional review board approval was acquired for this study. A retrospective review of operative logbooks was carried out to identify all patients who had partial MUS removal due to urethral erosion over a 7-year period by a single surgeon. A chart review of all patients was then conducted to ascertain the patients’ presenting complaint, type of MUS previously inserted (where available), initial investigations, the nature of the operative intervention required, post-operative outcomes, and follow-up. Timeline from initial operation to presentation with symptoms was sparsely available and therefore not evaluated.

All procedures were carried out by a single urological surgeon. Pre-operative diagnostic per-vagina examination and flexible cystoscopy allowed accurate operative planning by delineating the location of the MUS erosion and degree of calcification.

Cases with significant intra-urethral MUS calcification often required two operations. In the first instance, an endoscopic approach allowed use of a 365-µm holmium laser fiber using 10 Hz frequency and 1 J energy. The MUS calcification was completely fragmented and the visualized intra-urethral MUS was then laser ablated down to the site of erosion at the mucosal edge. Similarly, if the MUS was traversing the urethra in a “through and through” fashion, then an endoscopic approach was used to allow laser ablation of fibers to divide the MUS into two separate arms.

Definitive partial MUS removal was subsequently completed using a vaginal approach with an inverted-U incision to allow excision of the extra-urethral MUS component. This approach allowed for the removal of both arms as close to the inferior pubic ramus as was feasible in the majority of cases.

In cases that required formal urethral repair, a two-layer closure with interrupted 4–0 monofilament absorbable suture was used. An indwelling catheter was left in situ for 4 weeks post-operatively. All patients had only partial excision of the MUS erosion to the pubic bone to limit morbidity [3] of complete excision. The remainder of the trans-obturator or retropubic arms of the MUS were left in situ.

All excised material was sent for histopathological assessment and documentation. A flexible cystoscopy with urethral catheter removal was performed 4 weeks post-operatively, which permitted assessment of urethral integrity. All patients were advised to use topical vaginal oestrogen for a minimum of 6 weeks post-operatively to begin at time of discharge.

Data were collected on post-operative patient symptoms and details regarding subsequent surgery for SUI management if required. Patients were specifically questioned regarding resolution of irritative lower urinary tract symptoms/UTIs and new-onset symptoms post mesh removal. All cases were discussed at the local monthly mesh multi-disciplinary team meeting.

## Results

A total of 23 patients were identified between 2016 and 2023. Median age was 56 (range 40–77 years). Only patients with urethral MUS erosions were included in the analysis. Two patients had isolated vaginal erosions and presented with dyspareunia and hispareunia alone without urinary symptoms that were excluded from the analysis. The remaining 21 patients had urethral erosion of the MUS.

Two-thirds of patients presented with UTIs. Less common presentation was with overactive bladder symptoms (52%) and recurrent incontinence (42%). Some women presented with a combination of symptoms.

The initial MUS surgery comprised 9 patients who had undergone insertion of a trans-obturator tape (TOT), 6 patients with a trans-vaginal tape (TVT), and 2 patients who had undergone two previous MUS insertions. Four patients had an unspecified type of MUS where the original operative details were unavailable and the patient could not confirm the type of MUS.

A total of 18/21 (86%) of the patients with urethral MUS erosion required formal urethral repair after excision of MUS and a 4-week period with an indwelling catheter. Four patients had significant calcification of the intra-urethral MUS erosion and required the use of laser fragmentation.

All patients were reviewed in the clinic 8 weeks after catheter removal and their symptoms were evaluated. All patients who had reported pre-operative dyspareunia and UTIs had complete resolution of their symptoms post-operatively. The majority of patients (17/21; 81%) reported significant stress urinary incontinence. Of these, 42% (9/21) had experienced pre-operative SUI and the remainder (12/21) had new, de novo, SUI.

Fifteen of these 17 patients (88%) who reported SUI after excision of MUS erosion required one or more further interventions to manage their stress incontinence. This included autologous rectus fascial sling (ARFS) insertion (66%), intraurethral bulking agent injection (14%), or formation of a diverting ileal conduit (13%).

One patient initially underwent injection of an intraurethral bulking agent and subsequently proceeded to insertion of an ARFS due to ongoing symptoms.

No intraoperative complications occurred. No post-operative complication greater than Clavien-Dindo group 2 occurred.

## Discussion

Urethral MUS erosion is a relatively rare complication after MUS insertion affecting between 0.3 and 3.3% of patients [4–7]. The incidence is thought to be under reported as this complication can present with a number of non-specific symptoms and as a result there can often be a delay in diagnosis. In our cohort, 66% of patients presented with UTIs. The majority of these women had been managed in the community for a number of months prior to being referred for specialist opinion. Half (52%) of the patients presented with OAB symptoms, 42% recurrent stress incontinence, and 9% dyspareunia.

Sobota et al. [8] performed a systematic review of management of bladder and urethral mesh erosions. This study highlighted that the existing literature comprises small patient cohorts and single case reports in management of MUS erosion. Most common presenting symptoms were similar to our cohort with 56% presenting with UTI, 27% with irritative LUTs, and 31% with obstructive LUTS. Overall, 88% of their cohort had complete resolution or improved symptoms following mesh removal.

Little evidence, consensus, or guidance is available to decide on the best approach for management of these complex cases [9–11]. Various small series in the literature have described different techniques for removal including exclusive trans-vaginal approach [9], combination of trans-vaginal ± trans-abdominal [12], endoscopic with laser [3, 4] or endoscopic with TUR [3, 13], and laparoscopic approach [14]. From the review, it appears that more extensive excision is generally undertaken in patients with pain as their primary presenting symptom [4, 11, 14].

In our patient series, the aim of intervention was to relieve urinary symptoms with a minimally invasive technique to reduce morbidity. Only partial removal of mesh was performed with excision of the two arms just anterior to the pubic bone via a trans-vaginal approach. No attempt was made at excision of MUS past this point. Syan et al. [11] used a similar technique in their study of 34 women including those with vaginal exposure and urethral perforation. This group removed all accessible tape via the trans-vaginal approach route in 90% of their patients. However, in a case of bladder perforation, a trans-abdominal combination approach was utilised. Similarly, in 2 patients presenting primarily with pain, complete excision was felt necessary.

Allagany et al. [4] recently published on their patient series of 29 patients over 10 years who excised intra-urethral portion of mesh erosion using tissue ablation laser settings so that all mesh in the urinary system was removed. This group found no difference in outcomes for patients who had no remaining mesh on re-look cystoscopy vs those who had minute residual mesh. This group identified that 21% of their cohort had new or worsening SUI post-operatively. Ten percent of this group underwent subsequent trans-vaginal removal of remaining mesh due to ongoing vaginal pain. This was with a two-step operation that is similar to the technique in our patient cohort.

Karim et al. [3] reported on the role of endoscopic management in MUS erosion in their systematic review. This group compared endoscopic ablation of mesh using holmium laser against the use of a resecting loop or laparoscopic scissors. Overall, this group reported that laser approach allowed for decreased post-operative morbidity and complications, decreased inpatient stay and good functional outcomes.

A large proportion (81%) of our patients with urethral erosion reported stress urinary incontinence after excision of MUS. The majority of these patients had worsening of their pre-existing SUI; however, 38% of patients had new SUI. The recurrence of SUI varies in the literature [9] but is in line with our findings. Hermieu et al. [9] discussed the outcomes of their small patient series following removal of mid-urethral sling erosion. They found 86% had SUI after intervention. Of which, 84% of these women went on to have a further procedure for management of their recurrent SUI. Sabota’s [8] review reported 65% of women had recurrent SUI. Similarly, Duckett et al. [10], in their International Urogynecological Association (IUGA) review of the literature, found similar rates of SUI recurrence (57%) and reported that 46% of these women underwent further surgery including autologous fascial sling AFS, bulking agent, and colpo-suspension.

All patients in our cohort reporting new or persistent SUI after removal of MUS erosion were offered both conservative and operative management options. Operative management included intra-urethral bulking agent (polyacrylamide hydrogel) or autologous rectus fascial sling (ARFS) insertion. De-functioning ileal conduit was only offered to those failing or not suitable for either ARFS or intra-urethral bulking agent who had ongoing significant SUI.

ARFS has been shown to be a suitable salvage option in those who have failed other incontinence procedures, including those in whom a MUS has not improved their symptoms, or as in this series, in those who have had significant erosion of MUS necessitating excision [15, 16]. Multiple studies have shown favourable results in both concomitant [11, 12, 17] and delayed [11, 15, 16] ARFS following removal of MUS with or without erosion. These studies describe good success rates in improvement of SUI. McCoy et al. [17] offered concomitant autologous fascial pubovaginal sling placement after MUS removal to their 46 patient cohort. They had significant success rates with pre-operative SUI of 91% and post-operative SUI of 9%. Syan et al. [11] compared immediate versus delayed insertion of ARFS and found similar rates of resolution between the two groups (66.7% vs 71.4%); however, each group had less than 10 patients. No patient in our series was offered concomitant insertion of ARFS at the time of initial excision of erosion.

At this time, the insertion of MUS has been suspended in Ireland pending review. The National Institute for Health and Care Excellence (NICE) guidelines [18] advise that colpo-suspension, ARFS, and intramural bulking agent should be offered to any woman wishing to pursue operative intervention for management of their SUI.

The guidelines advise that a woman with new-onset symptoms following MUS insertion should be referred for specialist opinion. Women considering mesh removal should be extensively counselled and consented, and their expectations of treatment should be realistic. To that end, women undergoing MUS removal should be informed that complete MUS removal may not be possible; however, partial removal may relieve urinary symptoms but may not relieve pain symptoms. They should also be advise that surgery to remove mesh often results in recurrent stress incontinence and that they may require a further procedure to improve continence. The IUGA has made similar recommendations in their guidance document [10].

Limitations of our study include its retrospective nature and the lack of validated questionnaire utilisation. The patient series and reported outcomes in the literature are largely made up of small, retrospective patient cohorts without validated questionnaires. We welcome any consensus or guidance on the management of these complex cases. However, due to the heterogeneity of this patient population in terms of erosion location (vaginal, bladder, urethral), degree of calcification, and patient presenting symptoms, we feel that this will be difficult to achieve and that individualised operative approaches on a case-by-case basis are required for optimal results.

## Conclusion

Partial removal of MUS due to urethral erosion improves patient symptoms. However, the majority have recurrence of SUI and may require further intervention.
